# Mechanisms of Action of the Host-Targeting Agent Cyclosporin A and Direct-Acting Antiviral Agents against Hepatitis C Virus

**DOI:** 10.3390/v15040981

**Published:** 2023-04-17

**Authors:** Dandan Liu, Tanya P. Ndongwe, Juan Ji, Andrew D. Huber, Eleftherios Michailidis, Charles M. Rice, Robert Ralston, Philip R. Tedbury, Stefan G. Sarafianos

**Affiliations:** 1 CS Bond Life Sciences Center, Department of Molecular Microbiology & Immunology, University of Missouri, Columbia, MO 65201, USA; 2 CS Bond Life Sciences Center, Department of Veterinary Pathobiology, University of Missouri, Columbia, MO 65201, USA; 3 Laboratory of Virology and Infectious Disease, The Rockefeller University, New York, NY 10065, USA; 4 Laboratory of Biochemical Pharmacology, Center for ViroScience and Cure, Department of Pediatrics, Emory University School of Medicine and Children’s Healthcare of Atlanta, Atlanta, GA 30322, USA

**Keywords:** hepatitis C virus, NS5A, cyclophilin A, cyclosporin, host-targeting agents

## Abstract

Several direct-acting antivirals (DAAs) are available, providing interferon-free strategies for a hepatitis C cure. In contrast to DAAs, host-targeting agents (HTAs) interfere with host cellular factors that are essential in the viral replication cycle; as host genes, they are less likely to rapidly mutate under drug pressure, thus potentially exhibiting a high barrier to resistance, in addition to distinct mechanisms of action. We compared the effects of cyclosporin A (CsA), a HTA that targets cyclophilin A (CypA), to DAAs, including inhibitors of nonstructural protein 5A (NS5A), NS3/4A, and NS5B, in Huh7.5.1 cells. Our data show that CsA suppressed HCV infection as rapidly as the fastest-acting DAAs. CsA and inhibitors of NS5A and NS3/4A, but not of NS5B, suppressed the production and release of infectious HCV particles. Intriguingly, while CsA rapidly suppressed infectious extracellular virus levels, it had no significant effect on the intracellular infectious virus, suggesting that, unlike the DAAs tested here, it may block a post-assembly step in the viral replication cycle. Hence, our findings shed light on the biological processes involved in HCV replication and the role of CypA.

## 1. Introduction

Chronic hepatitis C virus (HCV) infection is a major cause of severe liver diseases, including liver cirrhosis and hepatocellular carcinoma, with an estimated 58 million people infected worldwide, and approximately 290,000 people dying from HCV in 2019 [1,2]. Until recently, the standard of care for HCV infection comprised pegylated interferon-alpha (pegIFN-α) and ribavirin (RBV), which elicit a sustained virologic response (SVR) of less than 50% in the most common genotype (genotype 1) viruses [3]. IFN/RBV therapy also gives rise to severe side effects, including fatigue, depression, and anemia, leading many patients to discontinue therapy [4]. This drove major efforts in the development of potent direct-acting antivirals (DAAs) that target essential HCV viral proteins. Drugs targeting the viral protease NS3/4A, the pleiotropic RNA binding protein NS5A, and the RNA-dependent RNA polymerase (RdRp) NS5B have been approved by the Food and Drug Administration (FDA), and their use in combination therapy is associated with high SVR rates, leading to cures in over 95% of the treated patients [5,6,7].

HCV is a *Hepacivirus* that possesses a positive single-strand RNA genome of ~9.6 kb. The genome is translated as a single polyprotein of ~3030 amino acids, which is proteolytically cleaved by cellular and viral proteases into ten mature viral proteins [8]. The viral structural proteins comprise a core and the envelope glycoproteins (E1 and E2), and the viral nonstructural proteins are the viroporin p7, the autoprotease NS2, the protease/helicase NS3/4A, the membrane-reorganizing NS4B, the multi-functional RNA binding phosphoprotein NS5A, and the RdRp NS5B [9].

NS5A is a protein consisting of approximately 450 amino acids that localizes to the endoplasmic reticulum (ER) and ER-derived membranes [10]. It forms a dimer [11,12,13] and has pleiotropic functions, including RNA binding activity required for efficient RNA replication [14]. A key function of NS5A is to drive the localization of replication complexes at lipid droplets (LDs) for viral assembly; LD localization depends on RNA binding, the PTPPL motif (residues 100–104), and diacylglycerol acyltransferase-1 [14,15,16,17]. NS5A comprises three domains (DI, DII, and DIII). DI and DII are indispensable for RNA replication [18,19], whereas DIII plays an important role in infectious virus assembly [20]. Both DII and DIII have been shown to interact with cyclophilin A (CypA) in order to regulate HCV RNA replication and virus production [21,22,23]. More recently, the modification of NS5A by interferon stimulated gene 15 (ISG15) has been suggested to enhance the interaction between NS5A and CypA [24]. Despite this progress, a complete understanding of the biological importance of the interactions between NS5A and CypA remains elusive.

Cyclophilin proteins are highly abundant, and cytosolic peptidyl-prolyl isomerases [25] are some of the most studied host factors in HCV replication. CypA has been shown to be essential for HCV replication in biochemical studies [26] in gene silencing experiments [27,28], as well as via the successful treatment of HCV-infected individuals with CypA inhibitors [29]; CypA compatibility has also been proposed to play a role in determining the host range of HCV [30]. An important inhibitor of CypA is the immunosuppressive drug cyclosporin A (CsA), which binds to CypA and inhibits the interaction with calcineurin [31]. The role of CypA in HCV replication, however, is unrelated to its function as an immune modulator, as demonstrated by the anti-HCV activity of CsA analogues which lack immunosuppressive properties [32,33], and may involve the peptidyl-prolyl isomerase activity of CypA, with NS5A as the substrate [23]. Although the activity of CypA has been associated with HCV NS2, NS5A, and NS5B proteins [34,35,36], NS5A appears to be the key binding partner for CypA [23] and CsA resistance mutations predominantly map to NS5A [27,37].

The first DAAs to be approved were boceprevir and telaprevir in 2011 [38,39]. Danoprevir (DNV) is a second-generation protease inhibitor with high specificity and antiviral potency, currently approved for use in China [40]. In 2013, the protease inhibitors were joined by sofosbuvir (SOF), a nucleoside analog targeting the HCV polymerase. The final class of anti-HCV DAAs to be approved comprised the highly potent NS5A inhibitors ledipasvir (LDV) and daclatasvir (DCV), approved by the FDA in 2014 and 2015, respectively [41,42,43,44]. They have been proposed to block RNA replication by acting on the replication complex (RC) and viral production by redistributing NS5A from the ER to LDs [45,46,47,48]. Although highly effective DAA regimens have been designed to combine one DAA with rapid antiviral effects with a second DAA with a high barrier to resistance, treatment failure due to viral resistance remained a problem, particularly for some HCV genotypes and for patients who failed prior therapy. This has in large part been addressed by the development of other more potent and better tolerated drugs and drug combinations, such as the NS3 protease and the NS5A inhibitor combination of glecaprevir–pibrentasvir, which can induce the SVR of 95% or higher with few side effects, leading to high adherence [49]. Similarly positive results have been described for combinations of sofosbuvir and the NS5A inhibitor velpatasvir [50,51,52], and triple therapy combining sofosbuvir–velpatasvir with the NS3 protease inhibitor voxilaprevir has proven to be efficacious for treatment-experienced patients [53,54]. Host-targeting antivirals (HTAs) have also been proposed as potential contributors to combination therapies, as they may provide a high genetic barrier to drug resistance, given the low rate of mutation of human genes compared to viral genes and the broad genotype coverage. The non-immunosuppressive CsA analogue alisporivir showed promise in clinical trials with pegIFN-α and RBV [55,56,57], although this promise has been surpassed by DAAs. Even without an immediate clinical need, it is nevertheless of interest to understand the essential roles played by host factors in viral replication.

In this study, we compared the inhibition kinetics, mechanisms of action, and antiviral efficacy levels of CsA to DAAs in Huh7.5.1 cells using a multiplex assay approach that we previously employed to illustrate the rapid anti-viral activity of NS5A inhibitors [58]. Our results show that CsA induced the rapid suppression of viral protein levels. In contrast, LDV was the fastest to suppress viral RNA levels. We found that NS5A inhibitors LDV and DCV rapidly suppressed both the infectivity of the intracellular virus and the extracellular virus, suggesting that they inhibit viral assembly. However, while CsA rapidly suppressed the infectivity of extracellular virus, it had no significant effect on the infectivity of the intracellular virus, indicating that it may block a step in the viral life cycle after assembly, but before, or at the step of, virus release. 

## 2. Materials and Methods

### 2.1. Cells and Viruses

Huh-7.5.1 cells have been previously described [59]. Cells were propagated in DMEM (Invitrogen), supplemented with 10% fetal bovine serum (FBS). Jc1-FLAG2(p7-nsGluc2A) [60], hereafter referred to as Jc1/Gluc2A, is a chimera of the J6 and JFH-1 HCV isolates and expresses *Gaussia* luciferase.

### 2.2. Compounds and Antibodies

DCV (BMS-790052) and DNV (RG7227) were purchased from Selleck Chemicals, (Houston, TX, USA). LDV (GS-5885) was purchased from MedChem Express (Monmouth Junction, NJ, USA). SOF (GS-7977) was purchased from Acme Bioscience (Palo Alto, CA, USA). Hoechst-33258 and BODIPY 493/503 were purchased from Invitrogen (Waltham, MA, USA), and HCS LipidTOX Deep Red Neutral Lipid Stain was purchased from Thermo Fisher Scientific (Waltham, MA, USA). Cyclosporin A (CsA—30024) was purchased from Sigma-Aldrich (St. Louis, MO, USA). Mouse monoclonal primary antibody 9E10, specific for NS5A, was provided by Brett Lindenbach (Yale University) [61]. Mouse monoclonal antibodies, specific for HCV core (C7-50), dsRNA (J2), and GAPDH (G-9), were purchased from Abcam (Waltham, MA, USA), Jena Bioscience (Jena, Germany), and Santa Cruz Biotechnology (Santa Cruz, CA, USA), respectively. Secondary antibodies Alexa 488-, 647- and HRP-conjugated anti-mouse IgG antibodies were purchased from Invitrogen and Santa Cruz Biotechnology, respectively.

### 2.3. The In Vitro Transcription and Electroporation of HCV RNAs

The Jc1/Gluc2A plasmid was linearized using XbaI and purified with Wizard SV Gel and the PCR Clean-Up System (Promega, Madison, WI, USA). Purified template DNA (1 µg) was transcribed using the MEGAscript T7 RNA production system (Ambion, Naugatuck, CT, USA). Template DNA was removed by treating it with Turbo DNase (Ambion, Naugatuck, CT, USA) at 37 °C for 15 min. RNA was cleaned up using the RNeasy Mini Kit (Qiagen, Germantown, MD, USA), and the RNA quality was monitored by agarose gel electrophoresis. RNA (10 µg) was electroporated into 5 × 10^5^ Huh-7.5.1 cells using 4 mm gap electroporation cuvettes (Thermo Fisher Scientific, Waltham, MA, USA) [(low range: 200, high range: 500, high CAP 500V Max, volts (kV): 0.27, high CAP (μF × 1000): 0.95)]. Electroporated cells were resuspended in pre-warmed DMEM plus 10% FBS and plated in T175 flasks.

### 2.4. Gaussia Luciferase Reporter Assay

Next, 2 × 10^5^ cells/well were plated in a 12-well plate. The following day, cells were infected with Jc1/Gluc2A virus at a multiplicity of infection (MOI) of 0.5 for 48 h. Then, 30 µL of supernatant was added to 30 µL of lysis buffer from the *Gaussia* Luciferase Glow Assay Kit (Thermo Fisher Scientific, Waltham, MA, USA) in black opaque 96-well microplates and incubated at room temperature for 20 min. Afterwards, 50 µL of 1× coelenterazine was added according to manufacturer’s instructions, and luciferase activity was measured with an EnSpire 2300 Multilabel Plate Reader (PerkinElmer, Waltham, MA, USA). To determine compound potency, serially diluted compounds were added to the cells prior to infection. Luciferase activity was then plotted against the log10 transformed drug concentration, and the concentration at which 50% reduction in viral replication was achieved (EC_50_) was determined using Prism (Graphpad).

### 2.5. Western Blot Analysis

Whole-cell extracts were prepared in the RIPA buffer (150 mM NaCl, 50 mM Tris/HCl pH 7.5, 1 mM EGTA, 1 mM EDTA, 0.1% SDS, and 1% TritonX-100) containing a cocktail of protease inhibitors (Sigma) and quantitated by the Bradford assay (Bio-Rad). Next, 50 μg of protein lysates was electrophoresed on an SDS–polyacrylamide gel and transferred to a polyvinylidene difluoride Immobilon-P membrane (Millipore). Membranes were probed with an anti-NS5A antibody (1:4000), an anti-core antibody (1:8000), or an anti-GAPDH antibody (1:5000), followed by an HRP-conjugated secondary antibody. Bound antibodies were visualized by adding Luminata Forte Western HRP substrates (Millipore, Temecula, CA, USA) to the membrane and imaging was conducted with a Fuji camera system. Protein quantities were then assessed by the densitometry analysis of band intensities using Image Gauge software (FujiFilm, Edison, NJ, USA).

### 2.6. Quantitative Reverse Transcription Quantitative PCR (RT-qPCR)

The total RNA sample was extracted with TRIzol (Sigma-Aldrich, St. Louis, MO, USA) according to the manufacturer’s instructions. The Power SYBR Green RNA-to-CT 1-Step Kit (Applied Biosystems, Foster City, CA, USA) was used to quantify the amount of HCV RNA. Primers specific for the 5′ UTR were 5’-TGCGGAACCGGTGAGTACA-3’ (forward) and 5’-TGCGGAACCGGTGAGTACA -3’ (reverse). The PCR cycling conditions were as follows: 30 min at 48 °C for reverse transcription, 10 min at 95 °C for enzyme activation, and 40 cycles of amplification with 15 s at 95 °C for denaturation and 1 min at 60 °C for annealing and extension. Standard curve reactions were run in parallel with serially diluted Jc1/Gluc2A plasmid, ranging from 2.0 × 10^7^ to 2.0 × 10^0^ copies.

### 2.7. Strand-Specific RT-qPCR

The initial reverse transcription step of the HCV 5′ UTR was carried out as previously described [62]. Briefly, 1 μg of RNA was denatured at 70 °C for 8 min with dNTPs and either the RC21 primer 5′-CTCCCGGGGCACTCGCAAGC-3′ (for the positive strand) or the tag-RC1 primer 5′-ggccgtcatggtggcgaataaGCCTAGCCATGGCGTTAGTA-3′ (for the negative strand), followed by incubation at 4 °C for 5 min. Thermoscript reverse transcriptase (Invitrogen) was added to the denatured RNA template and incubated at 60 °C for 1 h, followed by RNase H treatment for 20 min at 37 °C. Reverse-transcribed cDNA was mixed with RC1 (5′-GCCTAGCCATGGCGTTAGTA-3′) and RC21 primers for positive strand amplification and tag (5′-ggccgtcatggtggcgaataa-3′) and RC21 primers for negative strand amplification. Amplification was conducted by denaturation at 95 °C for 10 min, followed by 40 cycles of denaturation at 95 °C for 15 s and annealing/extension at 60 °C for 1 min using PerfeCTa SYBR Green FastMix (Quanta Biosciences, Gaithersburg, MD, USA). Amplification was carried out using the Applied Biosystems 7500 Fast Real-Time PCR Instrument.

### 2.8. Fluorescence Microscopy

Huh-7.5.1 cells were seeded into 8-well chambered coverglass (Thermo Fisher Scientific) at a density of 1 × 10^4^ cells/well. The cells were infected with Jc1/Gluc2A virus for 48 h before the virus-containing medium was replaced with the medium containing 100 × EC_50_ NS5A inhibitors, CsA, or the DMSO control. Infected cells were treated for 8 h before being fixed with 4% paraformaldehyde for 20 min and permeabilized with PBS supplemented with 0.1% Tween-20 (PBS-T) for 15 min. Cells were blocked with 1% BSA, 0.2% skimmed milk in PBS-T for 30 min, and stained with anti-NS5A antibody (1:1000) and dsRNA antibody J2 (1:1000) for 1 h. The secondary antibodies Alexa Fluor 488 goat anti-mouse IgG (H + L) and Alexa Fluor 647 goat anti-mouse IgG (H + L) (Invitrogen) (1:2000) were used to label the anti-NS5A antibody and the dsRNA antibody, respectively. The cells were counterstained with Hoechst-33258 to label the nuclei and/or BODIPY 493/503 (Invitrogen) or HCS LipidTOX Deep Red Neutral Lipid Stain (Thermo Fisher Scientific) to label LDs and mounted in the ProLong Gold Antifade Reagent (Life Technology, Carlsbad, CA, USA) or stored in PBS. Images were obtained using the Leica TCP SP8 MP confocal fluorescence microscope and the BioTek Cytation 5 multi-mode reader. 

### 2.9. Limited Dilution Assay (TCID50)

Next, 6 × 10^3^ cells/well were seeded into a 0.1% gelatin-coated 96-well plate. Cells were infected with 50 µL of six serial dilutions of virus-containing media, ranging from undiluted to 10^−^^5^ dilutions. Then, 72 h post infection, cells were fixed and permeabilized with 100% methanol for 30 min at −20 °C, washed with PBS followed by PBS-T, and blocked with 1% BSA and 0.2% skim milk in PBS-T. Afterwards, 3% hydrogen peroxide was added to block endogenous peroxidase activity. Cells were stained with anti-NS5A (1:25,000), ImmPRESS anti-mouse IgG (1:3000) (Vector Laboratories), and the 3,3’-diaminobenzidine tetrahydrochloride (DAB) substrate (1 drop/mL) (Invitrogen), respectively. NS5A-positive wells were counted and recorded using a light microscope. TCID50 was calculated with a Reed & Muench Calculator, as previously described [63].

### 2.10. Extracellular and Intracellular Infectivity Assays

Huh-7.5.1 cells were seeded into 12-well plates at 2 × 10^5^ cells/well. Cells were infected with the Jc1/Gluc2A virus for 48 h before the virus-containing medium was replaced with a 1 mL medium containing 100 × EC_50_ inhibitors or DMSO control and incubated at 37 °C for 8 h. For extracellular viral infectivity, 1 mL of supernatant was clarified by centrifugation (3000 × g) and transferred to 15 mL of disposable conical centrifuge tubes. The viral RNA was extracted from 200 μL of clarified supernatant using the QIAamp Viral RNA Mini Kit (Qiagen, Germantown, MD, USA), according to the manufacturer’s instructions, and was used to quantify the amount of released viral RNA by RT-qPCR. The remaining 800 μL of clarified supernatant was subjected to viral precipitation (to overcome the typically low titers of HCV produced from infected cells) by adding one quarter of the sterile-filtered 40% (*w*/*v*) PEG-8000 in PBS (final concentration is 8% (*w*/*v*)) and performing overnight incubation at 4 °C. Viral precipitates were collected by centrifugation (4000× *g*, 30 min) and washed twice with PBS. PBS supernatants were removed, and the pellets were resuspended in 1 mL of DMEM media containing 10% FBS. To determine the extracellular infectivity levels, naïve Huh-7.5.1 cells were infected with the HCV-containing media, normalized by the amount of released RNA (to ensure that the specific infectivity level was measured, not the assembly efficiency and the release), and a limited dilution assay was performed, as described above. For intracellular viral infectivity, infected cells were trypsinized, washed twice with PBS, and then resuspended in the medium. The cells were subjected to four freeze–thaw cycles using a dry-ice ethanol bath and a 37 °C water bath, and were then centrifuged to remove the cell debris. The supernatant was used to infect naïve Huh-7.5.1 cells and a limited dilution assay was performed, as described above.

### 2.11. Statistical Analysis

The data were collated and averaged, and standard errors of the means (SEMs) were calculated using Excel (Microsoft). Graphs were plotted and comparative statistics, typically using analysis of variance (ANOVA), were calculated using Prism (Graphpad).

## 3. Results

### 3.1. Time Course of HCV Inhibition by Antiviral Inhibitors

**Figure 1:**
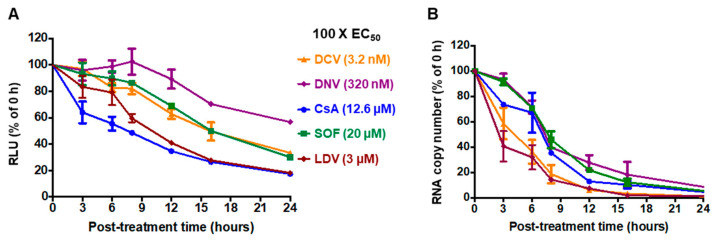
Time course of HCV inhibition by antivirals. Huh-7.5.1 cells were infected with Jc1/Gluc2A at an MOI of 0.5 for 48 h and then treated with 100 × EC_50_ of the indicated compounds. (**A**) Culture supernatants were analyzed for luciferase activity at the indicated time points. (**B**) The total cellular RNA sample was extracted using TRIzol reagent and analyzed for HCV 5′ UTR RNA by RT-qPCR at the indicated time points. The data were normalized to a DMSO-treated control. Each data point represents an average value of 3 individual experiments. The error bars represent the standard errors of the mean (SEMs). DCV, daclatasvir; DNV, danoprevir; CsA, cyclosporin A; SOF, sofosbuvir; LDV, ledipasvir.

To compare the inhibition mechanisms of CsA and DAAs, we first compared the compounds’ abilities to block HCV translation. Accordingly, Huh-7.5.1 cells were infected for 48 h with HCV (JC1/Gluc2A), and then were treated with one of the following antiviral inhibitors from distinct classes: the NS5A inhibitor DCV or LDV, the NS3/4A inhibitor DNV, the NS5B inhibitor SOF, or the CypA inhibitor CsA. The production of secreted luciferase from the HCV RNA was then measured over time. We performed these experiments using high concentrations (100 × EC_50_) of each compound where any reduction in viral protein would be pronounced and unambiguous. All compounds are expected to be non-cytotoxic at these doses; CsA, although a host-targeting compound, reportedly required a dose greater than 50 µM to induce 50% cell death in Huh7.5 cells [64]. A reduction in extracellular luciferase following CsA treatment was evident from 3 h post treatment (hpt), while LDV treatment led to a reduction from 8 hpt and matched the CsA response by 16 hpt (Figure 1A). The other inhibitors showed reductions after 8 hpt, although in the case of DNV, the effect was only minor (Figure 1A). Next, we compared the kinetic characteristics of antiviral inhibitors for their ability to suppress the relative levels of HCV RNA. Interestingly, LDV, DCV, and CsA exerted detectable reductions in RNA copy numbers at 3 hpt (Figure 1B). The fastest decrease was observed with the NS5A inhibitors; specifically, there were approximately 45% and 60% declines in HCV RNA copy number at 3 hpt with DCV and LDV, respectively, whereas there was approximately a 65% reduction in the HCV RNA copy number at 8 h following the addition of CsA (Figure 1B). Collectively, these results suggest that while CsA is the most rapid suppressor of protein expression, NS5A inhibitors act more rapidly to suppress viral RNA replication.

### 3.2. Kinetics of HCV Protein Suppression

**Figure 2:**
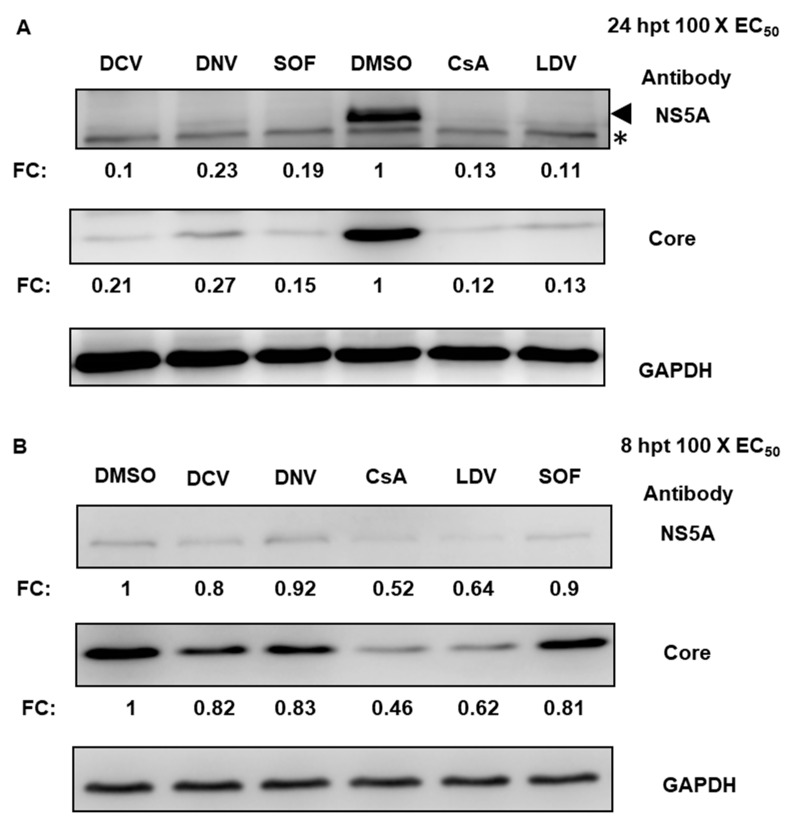
Suppression of HCV protein expression. Jc1/Gluc2A-virus-infected Huh-7.5.1 cells were treated with 100 × EC_50_ of DCV, DNV, SOF, CsA, or LDV. Cell lysates were harvested 24 hpt (**A**) or 8 hpt (**B**), separated by SDS-PAGE and immunoblotted for NS5A or core. NS5A and core intensities were normalized to GAPDH and quantified as a relative fold change (FC) with respect to DMSO-treated samples, as shown in the blots below. Each value represents the average value of 3 individual experiments. In (**A**), the arrow indicates NS5A and the asterisk indicates a non-specific host protein. NS5A and Core were probed on the same membrane, thus sharing a GAPDH control. DCV, daclatasvir; DNV, danoprevir; CsA, cyclosporin A; SOF, sofosbuvir; LDV, ledipasvir. * indicated non-specific band.

To confirm whether reduced luciferase activity reflected a reduction in the translation of HCV proteins, we conducted Western blot analysis to detect two viral proteins: HCV core and NS5A. At 24 hpt, all antivirals caused significant decreases in the amounts of HCV NS5A and core (based on densitometry analyses applied to Western blots), presumably following the suppression of replication (Figure 2A). At 8 hpt, the levels of HCV proteins in cells treated with DCV, DNV, or SOF were ~80 to 92% of those in DMSO-treated cells; however, the levels of NS5A and core proteins were appreciably decreased by 40 to 50% with CsA and LDV treatment (Figure 2B). Hence, in agreement with a reduction in HCV translation measured by the luciferase reporter assay, the results of Western blot for viral proteins indicate that CsA and LDV treatment rapidly suppressed HCV NS5A and core protein production. 

### 3.3. The Effect of CsA and NS5A Inhibitors on the Localization of NS5A at LDs

**Figure 3:**
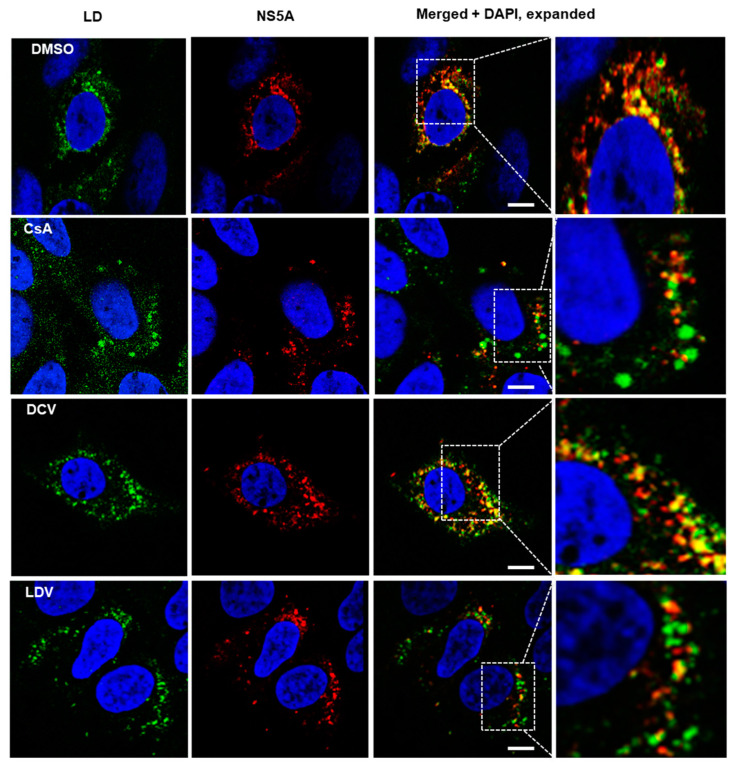
The distribution of NS5A and lipid droplets (LDs) following treatment with CsA and NS5A inhibitors. HCV-infected cells were treated for 8 h with DMSO, or 100 × EC_50_ DCV, CsA, and LDV. Cells were then fixed and stained with BODIPY 493/503 neutral lipid stain to label the LDs (green) and anti-NS5A antibodies (red), and with Hoechst-33258 to label the nuclei (blue). Images were acquired on a Leica TCS SP8 microscope with a 63× oil objective. Merged images and expanded merged images show the localization of NS5A and LDs. The scale bar represents 10 µm. DCV, daclatasvir; CsA, cyclosporin A; LDV, ledipasvir.

**Figure 4:**
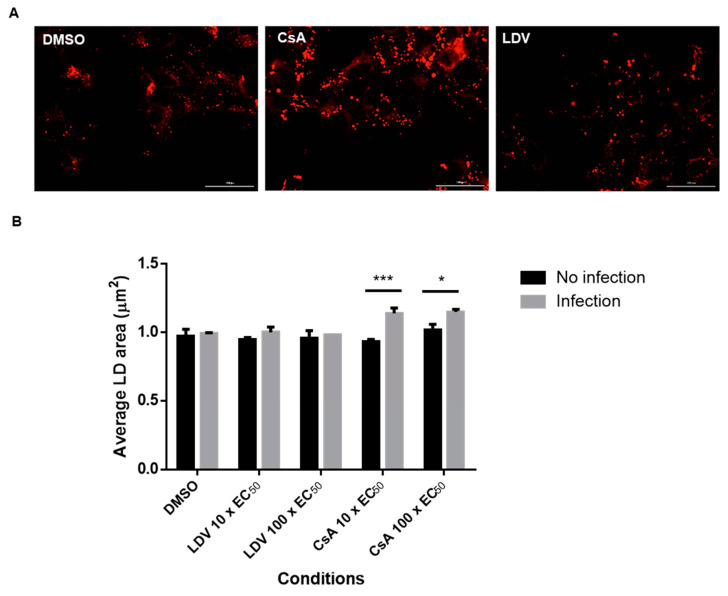
Changes in the LD area following treatment with CsA. Jc1/Gluc2A-virus-infected Huh-7.5.1 cells were treated with DMSO (10 × EC_50_ or 100 × EC_50_) of LDV or CsA for 8 h. The cells were fixed and stained with HCS LipidTox Deep Red Neutral Lipid Stain to label the LDs. Images were acquired on a BioTek Cytation 5 cell imaging multi-mode reader with 40× objective and analyzed by BioTek Gen5 software. (**A**) Representative images of LDs in cells treated with 100 × EC_50_ LDV or CsA for 8 h. The scale bar represents 100 µm. (**B**) The average area of LDs for each treatment. Each data point represents the average value of 2 individual experiments. The error bars represent the SEMs. Statistical analysis was performed using 2-way ANOVA with Šidák’s multiple comparisons test. * *p* < 0.05, *** *p* < 0.001. CsA, cyclosporin A; LDV, ledipasvir.

We and others have previously reported changes in NS5A localization following treatment with NS5A-targeting compounds [47,58]. To gain further insight into the impact of CypA inhibitors, HCV-infected cells were treated with DMSO, DCV, LDV, or CsA, and the distribution of NS5A and LDs was observed using confocal microscopy. Consistent with previous reports [47,58], the NS5A distribution appeared more punctate for all the drugs compared to DMSO (Figure 3). Furthermore, it appeared that the treatment of HCV-infected cells with CsA and LDV decreased the extent of NS5A co-localization with LDs (Figure 3). Notably, we also observed that the LDs were enlarged following treatment with CsA (Figure 3, expanded section). To quantify this effect, uninfected and HCV-infected cells were treated for 8 h with 10 × EC_50_ or 100 × EC_50_ CsA or LDV, LDs were stained with LipidTox Deep Red Neutral Lipid Stain (Figure 4A, red), automated unbiased imaging was performed, and the average area of LDs was calculated. In uninfected cells, no differences in the LD area were observed (ANOVA with Tukey’s multiple comparison test); however, the treatment of infected cells with CsA (but not LDV) induced a small but reproducible enlargement of LDs, suggesting that this change is specifically attributed to the inhibition of interactions between HCV and CypA (Figure 4B). These data suggest that CsA, but not LDV, caused LD enlargement, indicating different mechanisms of action.

### 3.4. Interaction between NS5A and HCV Double-Stranded RNA

**Figure 5:**
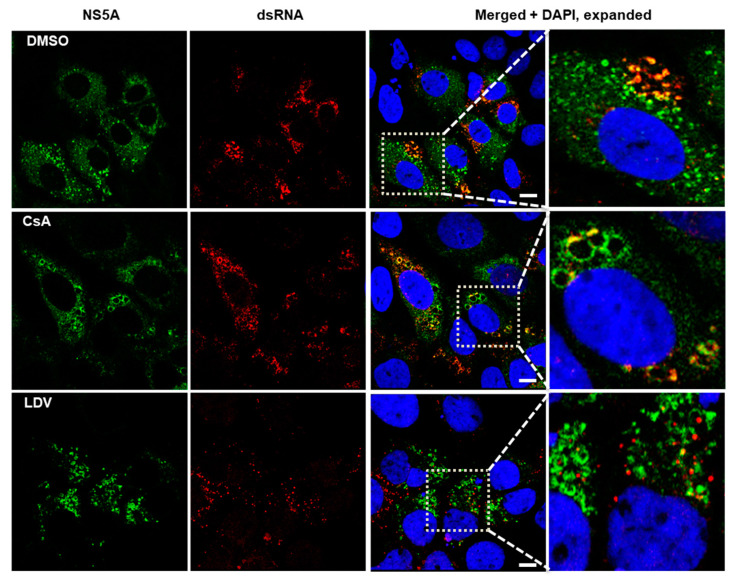
Localization of NS5A and dsRNA following treatment with CsA and LDV. Confocal immunofluorescence microscopy showing the cellular distribution of NS5A and dsRNA in HCV-infected cells treated for 8 h with DMSO (first row), 100 × EC_50_ CsA (second row), and LDV (third row). The cells were fixed and stained with 9E10 to label NS5A (green, first column), J2 to label dsRNA (red, second column), and DAPI to label nuclei (blue). Images were acquired on a Leica TCS SP8 microscope with a 63× oil objective. Merged images and expanded merged images show the localization of NS5A and dsRNA. The scale bar represents 10 µm. CsA, cyclosporin A; LDV, ledipasvir.

NS5A plays a critical role in HCV genome replication and assembly at LDs [14]. Double-stranded RNA (dsRNA) is formed during HCV replication and has been reported to co-localize with LDs and NS5A [65]. Using an antibody that recognizes dsRNA, we visualized HCV dsRNA and followed changes in its localization during treatment with LDV and CsA. We found that in cells treated with DMSO, NS5A was mostly diffused throughout the cytoplasm but was also observed on circular structures (presumably LDs) where it co-localized with dsRNA (Figure 5, expanded section). In cells treated with CsA, dsRNA mostly co-localized with NS5A at what appeared to be enlarged LDs (Figure 5, expanded section). Finally, in cells treated with LDV, dsRNA appeared to be restricted to discrete foci that did not co-localize with NS5A (Figure 5, expanded section). Collectively, these data suggest that CsA did not disrupt the co-localization of NS5A with dsRNA, although it may have perturbed the structure of lipid droplets. Conversely, LDV did appear to interrupt the interaction between NS5A and dsRNA.

### 3.5. Effects on HCV RNA Assessed by Strand-Specific RT-qPCR

**Figure 6:**
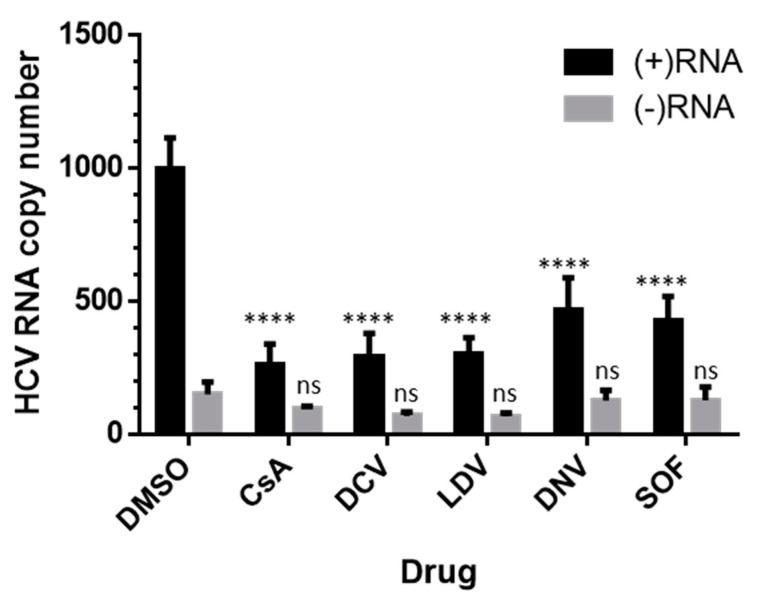
Strand-specific suppression of HCV RNA. Jc1/Gluc2A-virus-infected Huh-7.5.1 cells were treated with DMSO or 100 × EC_50_ of compounds for 8 h. Total cellular RNA was extracted by TRIzol reagent and analyzed for HCV 5′ UTR RNA by RT-qPCR. The cycle threshold (CT) values were converted to copy numbers based on the standard curve of the Jc1/Gluc2A-genome-encoded plasmid. The data are shown for drug effects on HCV (+)RNA (black) and HCV (−) RNA (grey). Each data point represents the average value of 3 individual experiments and the error bars represent the SEMs. Statistical analysis was performed using 2-way ANOVA with Tukey’s post hoc test to compare samples within the (+) RNA group or (−) RNA group. ns, not significant; **** *p* < 0.0001. DCV, daclatasvir; DNV, danoprevir; CsA, cyclosporin A; SOF, sofosbuvir; LDV, ledipasvir.

In order to dissect the inhibitory effects on HCV (+) or (−) strand RNA, we performed strand-specific RT-qPCR, as previously described [62,66]. Huh-7.5.1 cells were infected for 48 h and treated with DCV, LDV, DNV, CsA, or SOF at a 100 × EC_50_ concentration. All the inhibitors appeared to suppress HCV (+)RNA relative to the corresponding DMSO control (Figure 6). In contrast, the decreases in (−)RNA following the administration of inhibitors were less profound and not significant relative to DMSO (Figure 6). 

### 3.6. The Infectivity of Intracellular and Extracellular Viruses

**Figure 7:**
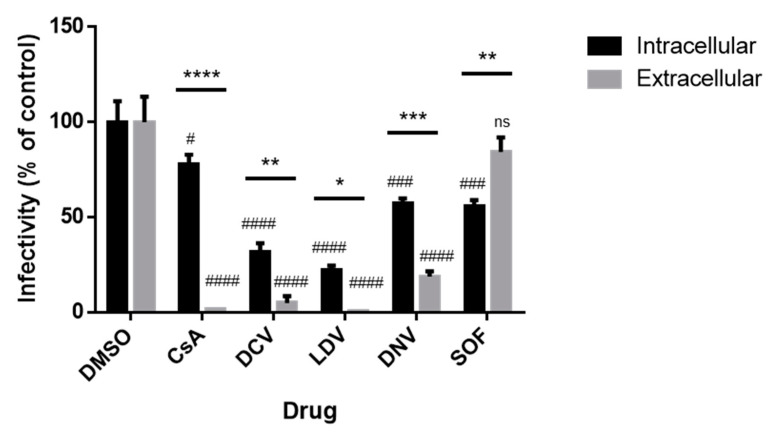
Impact of antivirals on intracellular and extracellular virus production. Jc1/Gluc2A-virus-infected Huh-7.5.1 cells were treated with DMSO or 100 × EC_50_ of antiviral inhibitors for 8 h. Intracellular virus was obtained from four cycles of freeze–thaw cell lysate, whereas extracellular virus was precipitated by PEG from culture supernatant. Viral RNA was extracted and the viral copy number was determined by RT-qPCR. Huh-7.5.1 cells were infected with a normalized amount of the virus and analyzed for viral titers by the limited dilution (TCID_50_) assay. Data were normalized to DMSO controls. Each data point represents the average value of 2 individual experiments. The error bars represent the SEMs. Statistical analysis was performed using 2-way ANOVA with Šidák’s multiple comparisons test to compare the effects on intra- vs. extracellular infectivity, *p*-values shown over bars: * *p* < 0.05; ** *p* < 0.01; *** *p* < 0.001; **** *p* < 0.0001. Analysis was also performed to compare compound treatments to corresponding DMSO controls. *p*-values indicate the columns above. ns, not significant; # *p* < 0.05; ### *p* < 0.001; #### *p* < 0.0001. DCV, daclatasvir; DNV, danoprevir; CsA, cyclosporin A; SOF, sofosbuvir; LDV, ledipasvir.

Finally, as we documented the effects of CsA and NS5A inhibitors on viral RNA and cellular LDs, as well as the site of virion assembly, we examined the effects of the inhibitors on HCV assembly and release. We performed limited dilution assays to assess intracellular and extracellular viral infectivity. Cells were infected with Jc1/Gluc2A for 48 h followed by 8 h of exposure to inhibitors at 100 × EC_50_ concentration. RNA was extracted from cell lysates or media. Following normalization based on the genome copy number, naïve Huh-7.5.1 cells were infected with these viral stocks, and viral infectivity was determined with a limited dilution assay after 72 h. We observed a significant reduction in both intracellular and extracellular viral infectivity (relative to the corresponding DMSO control) for all conditions except SOF (extracellular infectivity) (Figure 7). We additionally compared effects on intracellular versus extracellular infectivity and found that there was a significant difference in all cases. In the SOF-treated cells, intracellular viral infectivity was preferentially suppressed relative to extracellular viral infectivity, whereas for CsA and all other DAA treatments, extracellular viral infectivity was preferentially suppressed relative to intracellular viral infectivity (Figure 7); this effect was particularly strong (~50-fold) in cells treated with CsA. The ability of CsA to induce the strong inhibition of extracellular infectivity, without a corresponding impact on intracellular infectivity, suggests that CsA may act to impair the release of infectious viral particles.

## 4. Discussion

With the advent of DAAs that target essential HCV nonstructural proteins, tremendous progress has been made in developing highly effective combination therapies with low cost, high genetic barrier to resistance, and broad genotype coverage [7,51,52,53,54,56,57]. Therapeutics that target host cellular factors have the potential to increase the barrier to resistance even further. CypA is a host factor that has been the subject of great interest because it plays a critical role in HCV replication and is a potential binding partner of several HCV proteins. Hence, this component of basic virology is important in order to understand the role of CypA in HCV infection. Here, we examined the effect of CsA (presumed to be acting via CypA inhibition) on the HCV replication cycle and compared the mechanism of action of CsA to those of DAAs. 

We found that CsA exerted faster suppression of HCV protein expression than any DAA included in the study. In contrast, the NS5A-targeting compounds LDV and DCV induced the fastest suppression of HCV RNA levels. This suggests that CsA can act post-transcriptionally; this is because, at a given time point post treatment, CsA-treated cells contained more viral RNA, but less viral protein, relative to the effect on cells treated with DAAs.

We further pursued the mechanisms of CsA and NS5A-targetting drugs through studies on their interactions with LDs, an important site for HCV assembly and release [67]. Indeed, HCV release and specific infectivity were reduced following interference with apolipoprotein synthesis and secretion [68,69,70,71]; these similarities between the effects of interference with the apolipoprotein release pathway and those following CsA treatment suggest a potential mechanistic relationship. We found that CsA increased the LD size in infected cells and reduced NS5A co-localization with LDs. The NS5A inhibitor LDV also reduced NS5A co-localization with LDs, and additionally reduced dsRNA colocalization with NS5A, a phenotype consistent with reports that NS5A inhibitors can prevent NS5A-RNA binding [72], but that was not observed with CsA. NS5A inhibitors were found to suppress infectious virus levels in both intracellular and extracellular compartments, consistent with its activity against HCV replication and association with LDs. In contrast, CsA only suppressed the extracellular virus, consistent with the model that CsA impairs the interaction between NS5A and LDs, but not the interaction with dsRNA. These results suggest that the activity of CsA is related to NS5A, consistent with extensive reports on the CypA interactions with NS5A [13,18,21,22,23,24,73,74,75,76], but is distinct from the activity of NS5A inhibitors.

In recent years, many investigators have uncovered mechanisms by which CypA contributes to HCV replication. CypA interacts with NS5A to enhance its RNA binding activity, and that function is abolished by CsA [23,77]. More broadly, Cyp inhibitors suppress HCV replication by blocking the de novo formation of the membranous web (the NS4B rearranged cellular membranes upon which HCV replication takes place) [78,79,80] and displacing NS5B from the HCV RC [81]. Similarly, MxB has been reported to inhibit HCV replication by impairing the NS5A-CypA interaction [73,82]. Perhaps most pertinently, the non-immunosuppressive Cyp inhibitor NIM811 causes the accumulation of neutral lipids into LDs in the cell and decreases apolipoprotein B secretion through the VLDL pathway [83], as apolipoprotein secretion has been shown to play an essential role in HCV assembly and release; thus, these data provide a mechanism that is consistent with the observations presented here, whereby CypA may be an important factor for viral particle secretion.

The HCV genomic RNA has multiple functions: it is a replication template, an mRNA, and the packaged genome of infectious particles. The potential of CsA/CypA to influence HCV replication and the release of infectious particles is implied by two earlier studies. A S168A mutation in NS2 leads to the inhibition of HCV particle assembly, without affecting HCV RNA replication or protein synthesis; this assembly defect can be partially rescued through a mutation in NS5A V464L [84]. Intriguingly, the NS5A V464L mutation also confers the ability to replicate independently of CypA, thus allowing resistance to CsA [28]. These observations suggest that NS5A plays a key role in modulating the fate of HCV RNA, which agrees with extensive evidence demonstrating NS5A as a substrate for CypA [22,23,85,86] and the essential role of NS2 in particle assembly [84,87]. Together with our data, these observations are consistent with the close interdependency of genome replication and particle assembly in HCV, and the critical importance of CypA-NS5A interaction for the regulation of these processes.

In conclusion, we found that CsA both rapidly suppresses viral protein synthesis and potently inhibits the release of infectious HCV. While there is little demand for CsA-derived molecules as therapeutics for HCV, our findings support those of earlier studies and provide additional insight into the mechanism of action of CsA, and illustrate the virtues of exploiting pleiotropic proteins, such as NS5A, and virus–host interactions as antiviral targets.

## Data Availability

The data presented in this study are contained within the article.
